# Activation of the sweet taste receptor, T1R3, by the artificial sweetener sucralose regulates the pulmonary endothelium

**DOI:** 10.1152/ajplung.00490.2016

**Published:** 2017-09-28

**Authors:** Elizabeth O. Harrington, Alexander Vang, Julie Braza, Aparna Shil, Havovi Chichger

**Affiliations:** ^1^Vascular Research Laboratory, Providence Veterans Affairs Medical Center, Providence, Rhode Island; ^2^Department of Medicine, Alpert Medical School of Brown University, Providence, Rhode Island; ^3^Biomedical Research Group, Anglia Ruskin University, Cambridge, United Kingdom

**Keywords:** acute respiratory distress syndrome, artificial sweeteners, pulmonary endothelium, sweet taste, T1R3

## Abstract

A hallmark of acute respiratory distress syndrome (ARDS) is pulmonary vascular permeability. In these settings, loss of barrier integrity is mediated by cell-contact disassembly and actin remodeling. Studies into molecular mechanisms responsible for improving microvascular barrier function are therefore vital in the development of therapeutic targets for reducing vascular permeability in ARDS. The sweet taste receptor T1R3 is a G protein-coupled receptor, activated following exposure to sweet molecules, to trigger a gustducin-dependent signal cascade. In recent years, extraoral locations for T1R3 have been identified; however, no studies have focused on T1R3 within the vasculature. We hypothesize that activation of T1R3, in the pulmonary vasculature, plays a role in regulating endothelial barrier function in settings of ARDS. Our study demonstrated expression of T1R3 within the pulmonary vasculature, with a drop in expression levels following exposure to barrier-disruptive agents. Exposure of lung microvascular endothelial cells to the intensely sweet molecule sucralose attenuated LPS- and thrombin-induced endothelial barrier dysfunction. Likewise, sucralose exposure attenuated bacteria-induced lung edema formation in vivo. Inhibition of sweet taste signaling, through zinc sulfate, T1R3, or G-protein siRNA, blunted the protective effects of sucralose on the endothelium. Sucralose significantly reduced LPS-induced increased expression or phosphorylation of the key signaling molecules Src, p21-activated kinase (PAK), myosin light chain-2 (MLC2), heat shock protein 27 (HSP27), and p110α phosphatidylinositol 3-kinase (p110αPI3K). Activation of T1R3 by sucralose protects the pulmonary endothelium from edemagenic agent-induced barrier disruption, potentially through abrogation of Src/PAK/p110αPI3K-mediated cell-contact disassembly and Src/MLC2/HSP27-mediated actin remodeling. Identification of sweet taste sensing in the pulmonary vasculature may represent a novel therapeutic target to protect the endothelium in settings of ARDS.

## INTRODUCTION

Acute respiratory distress syndrome (ARDS) is a major cause of morbidity and mortality in patients suffering from several predisposing factors such as trauma, sepsis, and pneumonia. The syndrome occurs when vascular fluid and protein leak across the pulmonary microvascular endothelium into the alveolar air space, causing pulmonary edema formation, which is characteristic of the disease. Respiratory failure then occurs as a result of decreased gas exchange and lung compliance and initiation of inflammatory cascades (79). Thus a key hallmark of ARDS is permeability of the pulmonary microvascular endothelium to vascular fluid and protein.

Vascular permeability is regulated through several mechanisms depending on the stimulus; however, each mechanism results in the breakdown of cell-cell contacts and actin remodeling. The permeability of the monolayer occurs through disruption of cell-cell contacts, maintained by the adherens junction complex, and an increase in actin-myosin contractility (39, 40, 77). In the case of lipopolysaccharide (LPS), an endotoxin from gram-negative bacteria, endothelial permeability is mediated through its binding to Toll-like receptor 4. The resulting Src-dependent signaling cascade leads to phosphorylation of both VE-cadherin and myosin light chain-2 (MLC2) (65, 71). Furthermore, the expression of the heat shock protein families HSP27, HSP70, and HSP90 correlates with increased vascular permeability (5, 32, 36). Targeting of these molecular mechanisms has been shown to attenuate LPS-induced pulmonary edema formation in vivo (3, 15), indicating the potential role for these molecules in settings of ARDS.

Members of the bitter taste receptor family, and their signaling effectors, have been identified in pulmonary solitary chemosensory cells (SCCs) (24, 37, 62, 67, 76), ciliated epithelial cells (64), and smooth muscle cells lining the airways (20). In pulmonary smooth muscle, 21 of the 25 members of the bitter taste receptor family have been identified with bitter taste agonists leading to vasodilation and bronchodilation (10, 20). While studies have identified other members of the taste receptor family in SCCs, no functional output has been previously described (74). In recent years, sweet taste receptors have also been identified in extraoral locations, such as pancreatic β-cells, adipocytes, and cardiomyocytes (6); however, they have not been previously identified in the vasculature. Sweet taste is mediated by the G protein-coupled receptor (GPCR) T1R3, which can form a homodimer or a heterodimer with T1R2 (56). Sweet taste receptors are activated upon binding of intensely sweet molecules, such as artificial sweeteners, at low concentrations (<1 mM) or glucose at high concentrations (>300 mM) (44). The consumption of artificial sweeteners has increased in recent years, with the concentration in diet soda ranging from 150 to 500 µM (25). In humans, while the majority of artificial sweeteners consumed are excreted in faeces, a significant proportion are absorbed by the small intestine, identified within the circulation (plasma) and excreted in the urine as a nonmetabolized molecule (60, 72). Therefore, it is likely that, following consumption of a diet high in artificial sweeteners, the vasculature is exposed to high levels of these intensely sweet molecules.

In the studies presented here, we demonstrate, for the first time, the presence of the sweet taste receptor T1R3 in the pulmonary endothelium. Expression of the receptor was demonstrated to be modulated by barrier-disruptive agents; however, stimulation of T1R3 with the intensely sweet artificial molecule sucralose attenuates thrombin- and LPS-induced endothelial monolayer permeability. Furthermore, in vivo exposure to sucralose attenuates lung edema formation induced by *Pseudomonas aeruginosa*. Our studies show that sucralose-mediated protection of the endothelial barrier is dependent on the components of the sweet taste sensing pathway. Interestingly, exposure to high glucose does not protect the pulmonary endothelium. Finally, we implicate a role for HSP27, p110α phosphatidylinositol 3-kinase (p110αPI3K), MLC2, Src, and p21-activated kinase (PAK) in sucralose-mediated protection of the pulmonary endothelium. Our studies demonstrate that sweet taste sensing at the pulmonary endothelium plays a key role in barrier function. Stimulation of the sweet taste receptor may represent a novel target in the treatment of ARDS.

## METHODS

### 

#### Cell lines and reagents.

TRIzol and Superscript II (Invitrogen). Rat lung microvascular endothelial cells (LMVECs; Vec Technologies, Rensselaer, NY) were cultured in MCDB-131 media (Vec Technologies) and used between *passages 3* and *9*. LPS (endotoxin) from *Escherichia coli* serotype 011:B4, recombinant VEGF protein, and thrombin were purchased from Sigma-Aldrich (St. Louis, MO). The *P. aeruginosa* strain 103 (PA103) was a kind gift from Dr. Troy Stevens (University of South Alabama, Mobile, AL). Gustducin (*GNAT3*) and gustducin siRNA were purchased from Origene (Rockville, MD). T1R3 (*Tas1R3*) and Gαq siRNA were purchased from Santa Cruz Biotechnology (Santa Cruz, CA).

#### In vivo studies.

LPS or vehicle (saline) was administered to nonanesthetized, adult male 8- to 10-wk-old C57BL/6 mice via a single injection at different doses (1, 2.5, and 5 mg/kg ip). At 24 h after intraperitoneal injection of LPS or vehicle into mice, lungs were removed for homogenization. Untreated male Sprague-Dawley rats were euthanized at 8 wk, and both lungs and jejunal segments was isolated and stored in RNAlater (Thermo Scientific, Waltham, MA) at −80°C.

Mice were exposed to sucralose (1 g/kg) by oral gavage once a day for 1 wk. At the end point, live gram-negative bacteria *P. aeruginosa* (PA103) or PBS vehicle was administered via a single intratracheal injection (10^6^ colony-forming units. At 4 h after PA103 administration, wet and dry lung weights were taken.

All animal experimental protocols were approved by the Institutional Animal Care and Use Committees of the Providence Veterans Affairs Medical Center and Brown University and comply with the Health Research Extension Act and the National Institutes of Health guidelines.

#### RT-PCR.

Total RNAs were extracted from rat lung, jejunum and cultured LMVECs using the TRIzol reagent (Thermo Scientific, Waltham, MA) as per the manufacturer’s instructions. RNA was purified using the acid phenol/chloroform system and reverse transcribed using SuperScriptII (Thermo Scientific), and T1R3 transcripts were measured with β-actin (GenBank Accession No. NM_031 144; forward: 937-955 and reverse: 1,223-1,208) used as the housekeeping gene as described previously (8). Expression of the *Tas1r3* gene was measured using specific intron-spanning primers that were designed from the sequences published for rat (GenBank Accession No. NM_130818.1; forward: 2,107-2,126 and reverse: 2,327-2,308). Relative gene expression level was analyzed, for each sample, using the ΔCt method where ΔCt = (Ct_Tas1r3_ – Ct_β-actin_) corresponding to the detected threshold cycles for the target gene and β-actin control.

#### Western blot analysis.

LMVECs were exposed to LPS (1 µg/ml) or sucralose (0.1 mM) for 24 h. Cells were then lysed with RIPA buffer, resuspended in Laemmli buffer, and subjected to immunoblot analysis. Individual lobes of mouse lungs were homogenized in buffer [20 mM HEPES (pH 7.9), 1.5 mM NaCl, 0.25 M sucrose, 0.2 mM EDTA, 200 mM PMSF, 0.5 mM DTT, and 1.5 mM MgCl_2_] for 2 min and subjected to immunoblot analysis. Immunoblot analyses were performed on 10% SDS-PAGEs using a range of primary antibodies (Table 1) at a dilution of 1:1,000, except vinculin (1:5,000) and secondary antibody dilutions of 1:5,000. All samples were run on the same immunoblot for each protein analyzed. Antibody specificity verification was assessed based on previous publications (included in Table 1) or with siRNA knockout studies (see Fig. 5).

**Table 1.** List of antibodies used for protein phosphorylation and expression analysis by Western blot analysis

Antibody	Company	Phospho Site
Protein phosphorylation		
Phospho-Cofilin	Cell Signaling (30)	Serine 3
Phospho-MLC2	Cell Signaling (22)	Threonine 18/serine 19
Phospho-VASP	Cell Signaling (31)	Serine 239
Phospho-PAK 1/2	Cell Signaling (33)	Threonine 423/402
Phospho-Src	Cell Signaling (4)	Tyrosine 416
Phospho-ERK1/2	Cell Signaling (70)	Threonine 202/tyrosine 204
Phospho-p38	Cell Signaling (17)	Threonine 180/tyrosine 182
Phospho-p70 (T389)	Cell Signaling (78)	Threonine 389
Phospho-FAK (Y397)	Cell Signaling (12)	Tyrosine 397
Phospho-SHP2 (Y542)	Santa Cruz (12)	Tyrosine 452
Protein expression		
HSP90	BD Biosciences (43)	
FAK	BD Bioscience (12)	
HSP70	BD Biosciences (18)	
Cofilin	Cell Signaling (58)	
VASP	Cell Signaling (31)	
PAK1	Cell Signaling (4)	
MLC2	Cell Signaling (22)	
ERK1/2	Cell Signaling (35)	
p38	Cell Signaling (17)	
Src	Santa Cruz Biotechnology (34)	
SHP2	Santa Cruz Biotechnology (12)	
p70	Santa Cruz Biotechnology (26)	
T1R3	Santa Cruz Biotechnology	
β-Actin	Santa Cruz Biotechnology (2)	
Gαq	Santa Cruz Biotechnology	
p110αPI3K	Santa Cruz Biotechnology (82)	
Gustducin	Santa Cruz Biotechnology	
HSP27	Santa Cruz Biotechnology (63)	

PAK, p21-activated kinase; MLC2, myosin light chain-2; HSP, heat shock protein; p110αPI3K, p110α phosphatidylinositol 3-kinase.

#### Endothelial monolayer permeability.

Changes in endothelial monolayer permeability were assessed using the electrical cell impedance sensor technique (Applied Biophysics, Troy, NY), as previously described (16, 29). For analysis of monolayer permeability LMVECs were seeded to confluence onto collagen-coated electric cell-substrate impedance sensing arrays. For knockdown experiments, LMVECs were transiently transfected with T1R3, Gαq, or gustducin siRNA duplexes (300 nM) or ns, scrambled control, using the Amaxa (Allendale, NJ) electroporation technique as described previously (15). Monolayers were treated with either sucralose (0.1 mM), glucose (5.5, 11, and 25 mM), or vehicle (H_2_O) in the presence and absence of VEGF (50 ng/ml), thrombin (2 U/ml), LPS (1 µg/ml), or zinc sulfate (0.7 mM). Addition of treatments was made at the same time, and resistance was measured over time.

#### Statistical analysis.

For three or more groups, differences among the means were tested for significance in all experiments by ANOVA with Fisher’s least significance difference test. Significance was reached when *P* < 0.05. Values are means ± SD.

## RESULTS

### 

#### The sweet taste receptor T1R3 is expressed at the pulmonary endothelium.

**Fig. 1.**
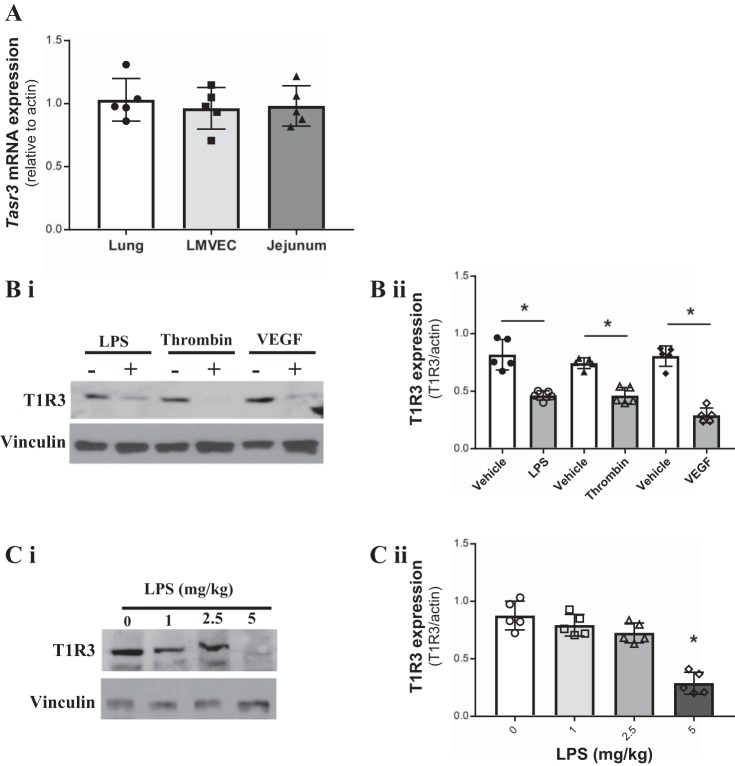
Expression of the sweet taste receptor T1R3 at the pulmonary endothelium is regulated by barrier disruptive agents. *A*: mRNA expression of the T1R3 gene *Tas1r3* in rat lung and jejunum tissue and cultured rat lung microvascular endothelial cells (LMVECs). Gene expression is relative to the housekeeping gene β-actin and normalized to the positive control jejunum tissue; *n* = 6. *B* and *C*: protein expression of T1R3 in cultured rat LMVECs exposed to LPS (1 µg/ml), thrombin (2 U/ml), or VEGF (50 ng/ml) for 24 h (*B*) and homogenates of lungs from C57/BL6 mice exposed to varying doses of LPS (0–5 mg/kg) (*C*); *n* = 5. A representative blot and densitometry relative to the load control (*i*) and β-actin (*ii*) are shown. Data are expressed as means ± SD. *P < 0.05 vs. vehicle.

The sweet taste receptor T1R3 is the key component of the sweet taste complex; T1R3 is necessary for the heterodimeric complex but can also form a homodimer for sweet taste sensing (19, 50, 56). In addition to the oral cavity, high expression of T1R3 mRNA (*TAS1R3*) and protein has been found in the small intestine, in particular the jejunum (41). mRNA expression levels of *TAS1R3* in rat lungs and LMVECs were comparable to the positive control rat tissue (jejunum) (Fig. 1*A*). To assess the link between sweet taste receptor and ARDS, protein expression of T1R3 was studied in LMVECs following exposure (24 h) to the barrier disruptive agents LPS, VEGF, and thrombin and in mouse lungs following exposure to LPS (4 h). In LMVECs, T1R3 protein levels were significantly reduced, to a similar degree, in the presence of all three agonists (Fig. 1*B*). Expression of T1R3 in mouse lungs was unaffected at low concentrations of LPS (1 and 2.5 mg/kg); however, at 5 mg/kg, lung injury and vascular leak were observed (13, 15), T1R3 expression was significantly reduced (Fig. 1*C*). These data demonstrate the presence of T1R3 in the lung microvasculature and implicates sweet taste sensing in endothelial barrier function.

#### The artificial sweetener sucralose attenuates barrier disruption in vitro and in vivo.

**Fig. 2.**
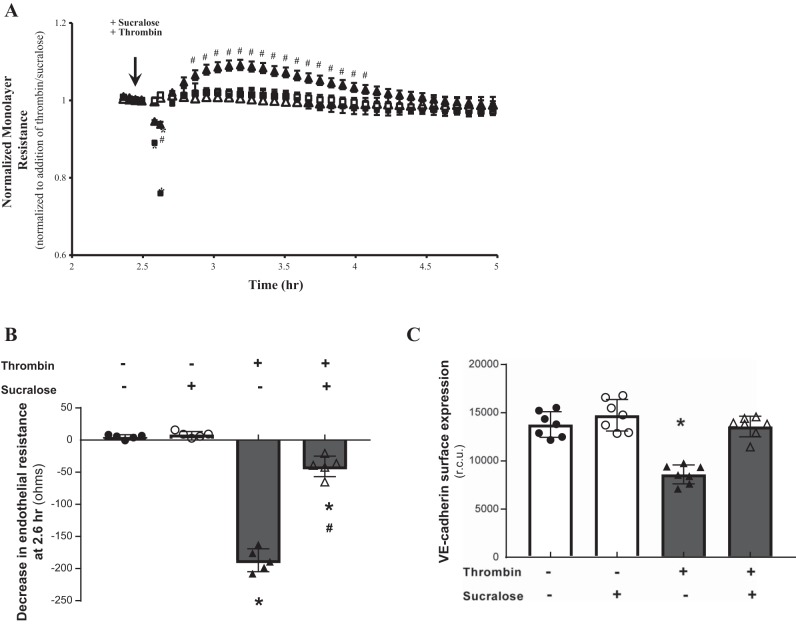
Stimulation of the sweet taste receptor with the artificial sweetener sucralose attenuates thrombin-induced barrier disruption and VE-cadherin internalization. *A* and *B*: changes in rat LMVEC endothelial monolayer resistance were measured using electrical cell impedance sensor in the presence (■, ▲) and absence (□, △) of thrombin (2 U/ml). Monolayers were exposed to sucralose (0.1 mM; ▲, △) or vehicle (H_2_O; ■, □) at the same time as thrombin. Permeability is shown as an experimental trace, normalized to the addition of thrombin and sucralose (*A*, arrow) and drop in endothelial resistance (*B*) measured at 12 min postthrombin and sucralose treatment; *n* = 5. *C*: cell surface expression of VE-cadherin was determined, with whole cell indirect ELISA using chemiluminescence, following exposure to thrombin and sucralose as per *A*; *n* = 6. Data are expressed as means ± SD. *P < 0.05 vs. vehicle for thrombin; #*P* < 0.05 vs vehicle for sucralose.

**Fig. 3.**
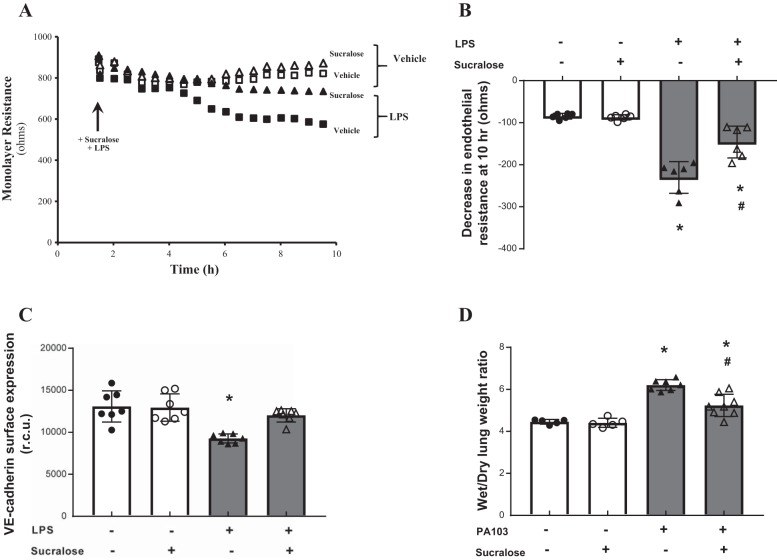
Stimulation of the sweet taste receptor with the artificial sweetener sucralose attenuates LPS-induced barrier disruption and VE-cadherin internalisation in vitro and bacteria-induced edema formation in vivo. *A* and *B*: changes in rat LMVEC endothelial monolayer resistance were measured using electrical cell impedance sensor (ECIS) in the presence (■, ▲) and absence (□, △) of LPS (1 µg/ml). Monolayers were exposed to sucralose (0.1 mM; ▲, △) or vehicle (H_2_O; ■, □) at the same time as LPS. Permeability is shown as an experimental trace, normalized to the addition of LPS and sucralose (*A*, arrow) and drop in endothelial resistance (*B*) measured at 10 h; *n* = 5. *C*: cell surface expression of VE-cadherin was determined, with whole cell indirect ELISA using chemiluminescence, following exposure to LPS and sucralose as per *A*; *n* = 6. *D*: lung edema formation was determined by measuring wet-to-dry lung weight ratio in mice following daily gavage of sucralose (1 g/kg) for 1 wk and 4-h exposure to *Pseudomonas aeruginosa* (PA103); *n* = 5–8. Data are expressed as means ± SD. *P < 0.05 vs. vehicle for LPS; #*P* < 0.05 vs vehicle for sucralose.

The sweet taste receptor complex is activated by low concentrations of intensely sweet molecules or high concentrations of sugars (51). Our previous studies demonstrate that endothelial permeability is closely related to adherens junction formation, with increased VE-cadherin surface levels observed in barrier protective settings (15). Therefore, we next assessed whether activation of T1R3 with the artificial sweetener sucralose at a concentration close to EC_50_ (51) has an effect on endothelial barrier function and VE-cadherin surface expression. LMVECs exposed to sucralose displayed no change in endothelial monolayer resistance (Figs. 2, *A* and *B* and 3, *A* and *B*) or VE-cadherin surface expression (Figs. 2*C* and 3*C*). Interestingly, thrombin-induced permeability and loss of VE-cadherin surface expression were significantly attenuated by concomitant exposure of LMVECs to sucralose (Fig. 2). Likewise, sucralose attenuated LPS-induced permeability and a decrease in VE-cadherin surface levels (Fig. 3). We next sought to establish whether sucralose exerted a protective effect on in vivo lung edema formation (wet-to-dry lung weight). Mice were exposed to a daily oral dose of sucralose over a 1-wk period, followed by exposure to *P. aeruginosa* (PA103) as a model for acute lung injury. Similar to in vitro findings, sucralose exposure significantly attenuated PA103-induced lung edema formation in vivo (Fig. 3*D*). Interestingly, sucralose exposure in the absence of PA103 had no effect on lung edema formation. Interestingly, both LPS- and thrombin-induced permeability in vitro and PA103-induced edema formation in vivo were not completely reversed by sucralose; however, the artificial sweetener did result in surface expression levels of VE-cadherin returning to baseline levels (Figs. 2 and 3).

**Fig. 4.**
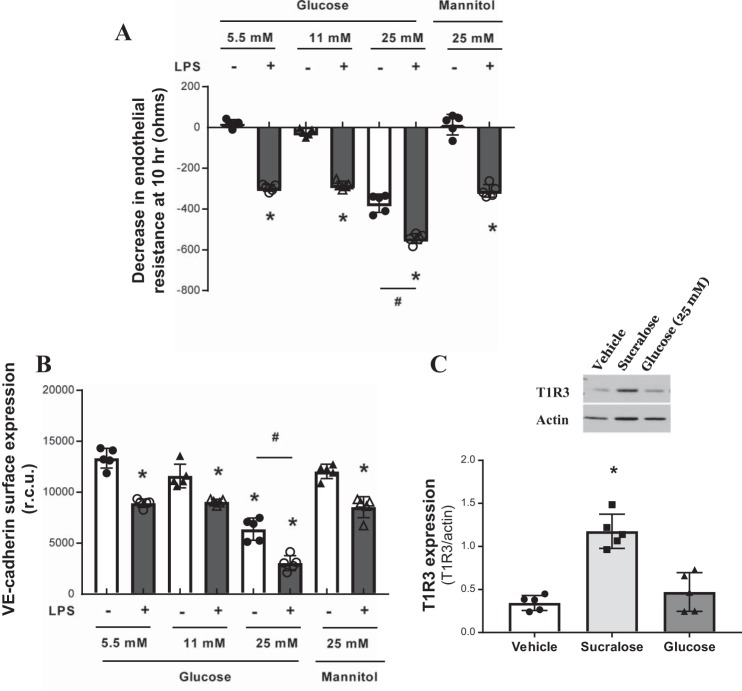
High-glucose exposure increases endothelial barrier permeability and VE-cadherin internalisation. *A*: changes in rat LMVEC endothelial monolayer resistance was measured using ECIS in the presence (closed bars) and absence (open bars) of LPS (1 µg/ml). Monolayers were exposed to different concentrations of glucose (5.5, 11, and 25 mM) or osmotic control mannose (25 mM) at the same time as LPS. Permeability is shown as drop in endothelial resistance measured at 10 h. *n* = 5. *B*: cell surface expression of VE-cadherin was determined, with whole cell indirect ELISA using chemiluminescence, following exposure to LPS and glucose as per *A*. *C*: protein expression of T1R3 in cultured rat LMVEC exposed to sucralose (0.1 mM), glucose (25 mM), or vehicle for both (H_2_O) for 24 h. A representative blot (*top*) and densitometry relative to the load control β-actin (*bottom*) are shown; *n* = 5. Data are expressed as means ± SD. *P < 0.05 vs. vehicle for LPS; #*P* < 0.05 vs. 5.5 mM control.

We next assessed whether glucose regulates endothelial barrier function in a similar manner. LMVECs were exposed to increasing concentrations of glucose from fasting levels (5.5 mM) to hyperglycemic levels (25 mM), with an osmotic control of mannose used for the high glucose concentration, in the presence and absence of LPS. High glucose (25 mM), but not lower glucose concentrations or mannose, significantly increased endothelial permeability and decreased VE-cadherin surface levels under baseline conditions (Fig. 4, *A* and *B*). LPS-induced permeability and decreased VE-cadherin surface levels were significantly exacerbated in the presence of high glucose but not lower glucose concentrations or mannose (Fig. 4, *A* and *B*). Interestingly, exposure of LMVECs to sucralose significantly increased protein levels of T1R3, while high glucose had no effect on expression of the sweet taste receptor (Fig. 4*C*).

Taken together, these data indicate that the intensely sweet molecule sucralose, but not high physiological levels of glucose, regulates T1R3 to protect the pulmonary endothelium against barrier disruption.

#### Barrier-protective effect of sucralose is mediated through sensing by the sweet taste receptor.

**Fig. 5.**
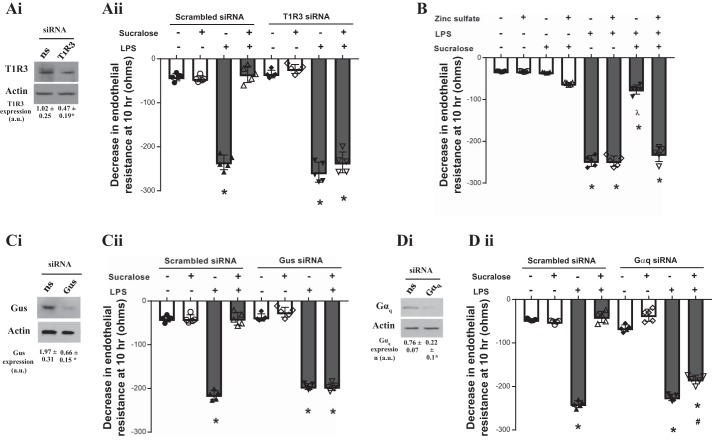
Barrier-protective effect of sucralose is mediated through sensing by the sweet taste receptor. *A*, *C*, and *D*: equivalent numbers of rat LMVECs were transiently transfected with scrambled (300 nM, open bars) or T1R3 (300 nM, closed bars) siRNA (*Aii*), gustducin (Gus, 300 nM, closed bars) siRNA (*Cii*) or Gαq (300 nM, closed bars) siRNA (*Dii*). After 48 h, changes in endothelial monolayer resistance were measured using ECIS in the presence and absence of LPS (1 µg/ml) and sucralose (0.1 mM). Permeability is shown as drop in endothelial resistance measured at 10 h (*ii*). Knockdown of endogenous protein was confirmed by immunoblot analysis of lysates from transiently transfected cells with an antibody specific to T1R3 (*Ai*), gustducin (*Ci*), and Gαq (*Di*). *B*: monolayer permeability was assessed in the presence and absence of the sweet taste inhibitor zinc sulfate (0.7 mM). Changes in endothelial monolayer resistance were measured using ECIS in the presence and absence of LPS (1 µg/ml) and sucralose (0.1 mM). Permeability is shown as drop in endothelial resistance measured at 10 h; *n* = 5–6. Data are expressed as means ± SD. *P < 0.05 vs. vehicle for LPS; ʎ*P* < 0.05 vs vehicle for sucralose; #*P* < 0.05 vs. LPS + vehicle for sucralose.

To study whether sucralose acts on the endothelial monolayer in a T1R3-dependent manner, the next experiments utilized inhibitors of the sweet taste receptor pathway. Molecular and chemical inhibition of T1R3 was performed using siRNA knockdown (Fig. 5*A*) and exposure to zinc sulfate (Fig. 5*B*), a chemical inhibitor of sweet taste receptor (23, 38). Endothelial permeability was assessed in the presence and absence of LPS and sucralose. Interestingly, attenuation of LPS-induced permeability by sucralose was significantly blocked by molecular (Fig. 5*Aii*) and chemical (Fig. 5*B*) inhibition of T1R3. In the presence of LPS alone, T1R3 inhibition had no impact on endothelial permeability (Fig. 5, *A* and *B*). Molecular inhibition of gustducin, a key signaling molecule downstream of T1R3 (53), was performed using siRNA knockdown (Fig. 5*Ci*). Knockdown of gustducin had no effect on endothelial permeability in settings of either LPS or sucralose exposure (Fig. 5*Cii*). Molecular inhibition of gustducin significantly abrogated sucralose-mediated protection of LPS-induced permeability (Fig. 5*Cii*). The G protein Gαq, which is highly expressed in the lung, has also been identified to play a role in sweet taste sensing (75, 80). Molecular inhibition of Gαq was performed in LMVECs using siRNA (Fig. 5*Di*). Protection of LPS-induced permeability, by sucralose, was reduced by 21% following knockdown of Gαq (Fig. 5*D*). These data indicate that sucralose exerts a protective effect on the endothelium in settings of barrier disruption through regulation of the sweet taste receptor and the downstream signaling pathway.

#### Sucralose attenuates LPS-induced elevated HSP27 and p110α and activation of MLC2, Src, and PAK.

**Fig. 6.**
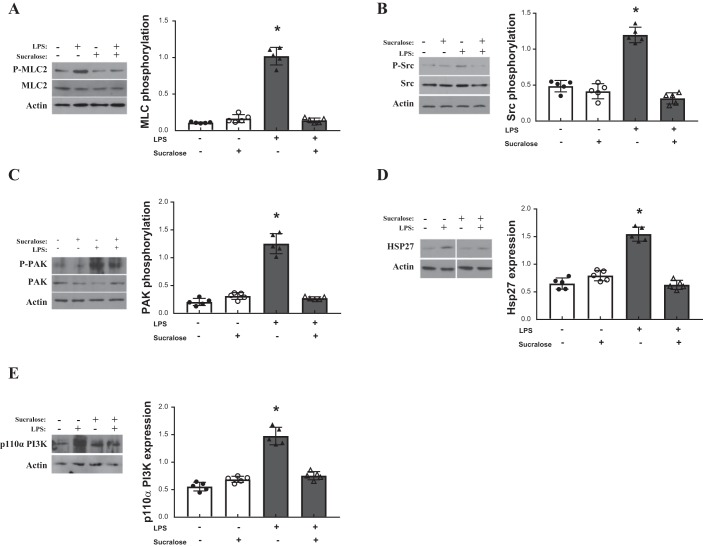
Sucralose attenuates LPS-induced elevated heat shock protein 27 (HSP27) and p110α phosphatidylinositol 3-kinase (p110αPI3K) and activation of myosin light chain-2 (MLC2), Src, and p21-activated kinase (PAK). Rat LMVECs were treated in the presence or absence of LPS (1 µg/ml) and sucralose (0.1 mM) for 24 h. Phosphorylation of MLC-2 (*A*), Src (*B*), and PAK (*C*) was assessed in whole cell lysates by immunoblot analysis with an antibody specific to each phosphorylated protein. Blots were stripped and reprobed for total protein expression and actin as a loading control. Total protein expression of HSP27 (*D*) and p110αPI3K (*E*) was also assessed in whole cell lysates, followed strip and reprobe of blots for actin as a loading control. Representative blots are shown. Nonessential lanes from the HSP27 representative blot (*D*) have been removed; *n* = 6. Data are expressed as means ± SD. *P < 0.05 vs. vehicle for LPS.

**Fig. 7.**
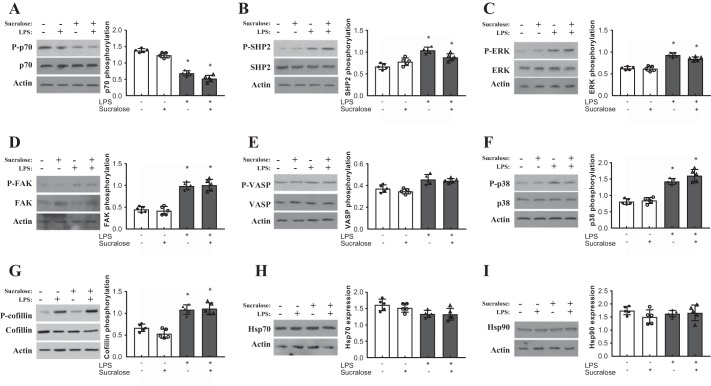
Role of sucralose on LPS-mediated signaling is independent of several key molecules. Rat LMVECs were treated in the presence or absence of LPS (1 µg/ml) and sucralose (0.1 mM) for 24 h. Phosphorylation of HSP70 (*A*), SHP2 (*B*), ERK (*C*), FAK (*D*), VASP (*E*), p38 (*F*), and cofillin (*G*) was assessed in whole-cell lysates by immunoblot analysis with an antibody specific to each phosphorylated protein. Blots were stripped and reprobed for total protein expression and actin as a loading control. Total protein expression of HSP70 (*H*) and HSP90 (*I*) was also assessed in whole-cell lysates, followed strip and reprobe of blots for actin as a loading control. Representative blots are shown. *n* = 6. Data are expressed as means ± SD. *P < 0.05 vs. vehicle for LPS.

To assess the molecular mechanism through which sucralose exerts an effect on LPS-induced signaling, key regulators of the adherens junction and endothelial barrier function were assessed for expression and activity. Phosphorylation of kinases FAK, p38, ERK, PAK, p70 and Src (15, 28); phosphatase SHP2 (14); filament proteins VASP and cofilin (59, 67); and MLC2 (7) were measured at phosphorylation sites relevant to protein activity (Table 1). Expression of heat shock proteins HSP27, 70, and 90 (11, 36, 45) and p110αPI3K (9) were also assessed. Sucralose treatment in the absence of LPS had no effect on phosphorylation or expression of any regulator molecule (Figs. 6 and 7). Phosphorylation of MLC2, Src, and PAK by LPS was significantly attenuated by exposure to sucralose (Fig. 6, *A*–*C*), whereas phosphorylation of other key regulators was unaffected by sucralose (Fig. 7, *A*–*G*). Unlike MLC2 and Src, in the presence of sucralose and LPS phosphorylation of PAK did not return to baseline conditions (Fig. 6, *A*–*C*). Expression levels of HSP27 and p110αPI3K were increased following exposure to LPS; however, this effect was abrogated by sucralose (Fig. 6, *D* and *E*). This effect was not observed in the other heat shock proteins, HSP70 and 90 (Fig. 7, *H* and *I*). Taken together, these data indicate that sucralose may attenuate LPS-induced permeability through inhibition of key barrier disruptive signaling molecules.

## DISCUSSION

In the present study we demonstrate, for the first time, the localization and function of the sweet taste receptor at the pulmonary endothelium. Our research identified the expression of T1R3 in the lung and microvascular endothelial cells, with reduced protein levels in response to the barrier-disruptive agents LPS, thrombin, and VEGF. We observed that activation of T1R3 by exposure to the artificial sweetener sucralose protects the microvasculature in vitro and in vivo against barrier disruptive agents through a sweet taste receptor-dependent pathway. Lastly, we implicated a role for sucralose in attenuating LPS-mediated Src, PAK, MLC2, HSP27, and p110αPI3K signaling. Therefore, the stimulation of T1R3 by artificial sweetener sucralose represents a novel mechanism through which the pulmonary microvasculature is regulated.

The sweet taste receptor T1R3 was first identified at the *Sac* genetic locus, which regulates sweet taste sensitivity, with expression observed in a subset of taste cells within the oral cavity (52, 54). Interestingly, T1R3 has recently been identified in extraoral locale, including the pancreatic β-cell, adipocytes, and the bladder; however, to date, no studies have assessed T1R3 in the vasculature (23, 55, 66, 73). Our study identifies T1R3 mRNA expression in the rat lung and microvascular endothelial cells. Interestingly, the mRNA levels of both were similar to those observed in the jejunum segment of the small intestine. Studies by others and us have identified T1R3 in different cell types within the small intestine, predominantly the jejunum, where activation of the receptor is linked to altered glucose metabolism in patients with metabolic diseases (8, 47, 49, 69). Therefore, T1R3 expression in the pulmonary vasculature is likely to be at a physiologically significant level. Endothelial cell protein expression of T1R3 was reduced by the barrier-disruptive agents LPS, thrombin, and VEGF. This was mirrored in the mouse lung where decreased T1R3 levels were noted following LPS treatment. Interestingly, at low doses of LPS, where no pulmonary edema is observed (data not shown), T1R3 expression is not significantly affected; however, at the 5 mg/kg dose, where pulmonary edema is observed (13, 15), T1R3 expression was significantly reduced. Therefore, it is likely that T1R3 expression plays a role in pulmonary endothelial barrier maintenance in vivo and in vitro. Indeed, following exposure to sucralose, which activates T1R3, barrier permeability caused by LPS and thrombin was attenuated. These findings were mirrored in an in vivo model of lung injury (*P. aeruginosa*), with sucralose exposure blocking lung edema formation. The in vitro protective role of sucralose was blocked following inhibition of sweet taste sensing, either by acting on the receptor via zinc sulfate (23, 38) or siRNA knockdown of T1R3 or downstream G proteins gustducin and Gαq (53, 75). Inhibition of T1R3 through siRNA or zinc sulfate attenuated the protective effect of sucralose on the endothelial barrier. The artificial sweetener is therefore acting through the sweet taste receptor to initiate a protective signaling response. Interestingly, Gαq inhibition did not completely blunt sucralose-mediated protection as seen in gustducin inhibition. It is therefore likely that gustducin, but not Gαq, is essential for T1R3-mediated signaling in the pulmonary endothelium.

Signaling mechanisms mediated by activated T1R3 vary depending on the cell type. In the pancreatic β-cell sweetener T1R3, binding results in insulin release mediated by elevated intracellular calcium levels (55) while in the adipocyte, Akt phosphorylation was noted to play a role in the stimulation of adipogenesis (66). In the pulmonary endothelium, studies presented here link the phosphorylation of Src, PAK, and MLC2, and increased expression of HSP27 and p110αPI3K, with sucralose-mediated protection from LPS. Previous studies have indicated a key role for Src and PAK phosphorylation, and p110αPI3K expression, in the breakdown of the pulmonary endothelium through dissolution of the adherens junction (9, 12, 15, 28) and in HSP27- and MLC2-mediated actin remodeling associated with barrier disruption (27, 32, 65, 71). Interestingly, other key regulators of the pulmonary endothelium, such as the filament proteins cofillin and VASP (59, 67), are phosphorylated by LPS but unaffected by sucralose. Thus, upon activation, T1R3 acts on a limited range of signaling molecules to regulate endothelial barrier function. The link between signaling downstream of T1R3 and Src/PAK/p110αPI3K and HSP27/MLC2 is unclear at present; however, it is possible that PLCβ2 recruitment, following the release of gustducin and Gαq, triggers the activation of kinases such as the inhibitory Src kinase Csk (48, 81). This in turn may regulate downstream molecules to protect the endothelial barrier from LPS-induced disruption. However, further studies are necessary to identify and understand the molecular mechanisms through which T1R3 downstream signaling regulates Src/PAK/p110αPI3K and HSP27/MLC2 within the pulmonary endothelium.

Sucralose is an intensely sweet, commercially available artificial sweetener with an estimated “sweetness” index of 600 times compared with sucrose (57). Sucralose, like many artificial sweeteners, stimulates the sweet taste receptor at low concentrations (<1 mM) (44). At glucose concentrations needed to stimulate T1R3 (>300 mM), endothelial cells are not viable due to hyperosmolarity (1, 21). We demonstrate that at a physiologically relevant high concentration of glucose (25 mM) vascular permeability was increased. Similar to previous studies, we also observed that high glucose exacerbates LPS-mediated barrier disruption (46); therefore, the protective effect of T1R3 activation with sucralose cannot be mimicked by glucose. Furthermore, while this study focused on the use of sucralose to activate T1R3, different artificial sweeteners demonstrate varying ability to bind T1R3 and stimulate downstream signaling (56). It is therefore possible that the level of pulmonary barrier protection exhibited by the sweetener is dependent on the type of sweet molecule used.

There is significant controversy regarding the benefit of artificial sweetener consumption in the diet. At present, a large proportion of the population consumes artificial sweeteners, such as sucralose, at high levels (25); however, clinical studies do not record any pulmonary responses in this population. Interestingly, our studies show that exposure of the microvasculature to sucralose in the absence of LPS has no effect on barrier function or on the expression or activation of key signaling molecules that regulate the endothelium. Therefore, it is possible that stimulation of T1R3 by artificial sweeteners only plays a physiological role in settings of vascular permeability. This represents the potential for artificial sweeteners to act as a novel therapeutic agent in diseases such as ARDS; however, further studies are necessary to assess the long-term effect of artificial sweeteners on the pulmonary vasculature. While the present study only assessed T1R3 expression, as it is the predominant sweet taste receptor that homodimerizes to sense sweet molecules, T1R2 can form a heterodimer with T1R3 and form a sweet taste receptor complex (56). Furthermore, T1R3 can heterodimerize with T1R1 to form an umami taste receptor complex. As our study demonstrates, a significant protective effect played by the sweet taste receptor in the pulmonary endothelium, it would be interesting to assess other taste-sensing complexes within the vasculature. In fact, previous studies have implicated that stimulation of the bitter taste receptor family (T2R) in airway smooth muscle and epithelial cells with bitter taste agonists stimulates bronchodilation and ciliary beat frequency, respectively (20, 64). Bitter agonists are currently under scrutiny as a treatment for asthma and chronic obstructive pulmonary disorder patients (42, 61, 68); however, studies are yet to assess the presence or activation of bitter taste receptors within the pulmonary vasculature. Our studies demonstrate that sweet taste agonists block the barrier disruptive effects of LPS on the pulmonary endothelium. There is therefore the potential for taste agonists to play a major role in various lung diseases in the future.

## GRANTS

This material is based on work supported by Diabetes UK Grant 15/0005284, Wellcome Trust Grant 202624/Z/16/Z, and American Heart Association Grant 13POST16860031 (to H. Chichger). E. O. Harrington was supported by National Heart, Lung, and Blood Institute Grants R01-HL-67795 and R01-HL-123965 and an Institutional Development Award (IDeA) under National Institute of General Medical Sciences Grant P20-GM-103652. A. Vang was supported by National Heart, Lung, and Blood Institute Grant 1R01HL128661.

## DISCLAIMERS

The views expressed in this article are those of the authors and do not necessarily reflect the position or policy of the Department of Veterans Affairs.

## DISCLOSURES

No conflicts of interest, financial or otherwise, are declared by the authors.

## AUTHOR CONTRIBUTIONS

E.O.H., A.V., A.S., and H.C. interpreted results of experiments; E.O.H. and H.C. edited and revised manuscript; E.O.H. and H.C. approved final version of manuscript; A.V., J.B., A.S., and H.C. performed experiments; A.V., A.S., and H.C. analyzed data; A.S. and H.C. prepared figures; H.C. conceived and designed research; H.C. drafted manuscript.
